# Evaluation of Different Combined Regimens in the Treatment of Cholinergic Urticaria

**DOI:** 10.1097/WOX.0b013e31825a72fc

**Published:** 2012-08-15

**Authors:** Abdulghani M Alsamarai, Ali A Hasan, Amina H Alobaidi

**Affiliations:** 1 Department of Medicine, Tikrit University College of Medicine, Asthma and Allergy Centre, Tikrit Teaching Hospital, Tikrit, Iraq 12112

**Keywords:** cholinergic urticaria, antihistamines, cimetidine, maprotiline HCl, chlorodiazopoxide, clindium bromide

## Abstract

**Background:**

Cholinergic urticaria is uncommon and accounts for 10% of all young adults. To date, there is no effective therapy for cholinergic urticaria.

**Objective:**

To determine the therapeutic efficacy of different drug combinations in the treatment of cholinergic urticaria.

**Patients and Methods:**

The participants included in the study are in the age range of 16 to 29 years, with cholinergic urticaria of any duration as diagnosed by physicians. Patients were recruited from Asthma and Allergy Centers in Baghdad and Tikrit. The selected patients were divided randomly into 3 groups according to the treatment protocol. All patients completed screening before treatment.

**Results:**

The study indicated that cholinergic urticaria was completely controlled in 30.4% of patients (group A) receiving 4 mg of chlorpheniramine maleate, half hour before the exercise, plus chlordiazopoxide (5 mg) and clindium bromide (2.5 mg) tablets, 3 times daily. However, cure rate was higher (83.1%) in patients (group B) receiving 4 mg of chlorpheniramine maleate (histadine), 3 times daily, plus 25 mg of maprotiline HCl (ludiomil), once daily at night. Furthermore, the complete cure rate was 85.4% in patients (group C) receiving 4 mg of chlorpheniramine maleate (histadine)3 times daily, plus 200 mg of cimetidine (tagadine), 3 times daily. The frequency of relapse was higher in group A (89%) as compared with group B (68.4%) and group C (23.5%) (*P <*0.0001).

**Conclusions:**

Combination of H1 and H2 antagonists was more effective based on complete control of cholinergic urticaria with lower relapsing rate. However, a future placebo-controlled clinical trial taking in consideration higher H1 antagonists than we used is warranted.

## 

Cholinergic urticaria is a very distinctive type of urticaria, in which characteristic small weal or/and itching occur. The prevalence of cholinergic urticaria is variable. Moore-Robinson and Warin[1] found that about 0.2% of patients in an outpatient dermatologic clinic had cholinergic urticaria. However, many published series have found cholinergic urticaria to be common. The prevalence of cholinergic urticaria is definitely higher in persons with urticaria; cholinergic urticaria affected 11% of a population with chronic urticaria in one study and 5.1% of persons with urticaria in another study [2,3].

Cholinergic urticaria is one of the physical urticaria brought on by a physical stimulus. Although the physical stimulus that triggers the cholinergic urticaria might be considered to be the heat, the actual precipitating factor is sweating. The definition and diagnostic testing of cholinergic urticaria has been the subject of consensus panel recommendations [4].

Mast cells seem to be critically involved in cholinergic urticaria [5]. Serum histamine, the principal mediator, rises in concentration with experimentally induced exercise, accompanied by eosinophil and neutrophil chemotactic factors and tryptase. A reduction of the alpha-1-antichymotripsin level, as seen in some other forms of urticaria, is present, and the eruption is improved with danazol. These findings have prompted some to argue for protease role in histamine release [6].

Although mast cells' release seems to be involved in cholinergic urticaria, less eosinophilic major basic protein is present than that in many other forms of urticaria [3]. The prevalence of cholinergic urticaria is variable, with a range from 0.2 to 11% [1]. Cholinergic urticaria occurs in both men and women, but it seems to be more common in men than in women, and occurrence usually begins in people aged 10 to 30 years, with an average age at onset of 16 years [7-10].

In cholinergic urticaria, the treatment goal is to ensure rapid and prolonged control of symptoms and a rapid return to normal social activities. Nonsedating H1 receptor antagonists, such as cetirizine, are the primary treatment modality. UV light has been beneficial in some patients with cholinergic urticaria, but there are contraindications to UV light. Ketofen may be helpful in patients with both cold urticaria and cholinergic urticaria. Danazol[11,12] and beta-blockers, such as propranolol, have been reported to be useful in cholinergic urticaria [13]. Benzoyl scopolamine administered topically and scopolamine butylbromide administered orally may be helpful in blocking the appearance of cholinergic urticaria lesions after challenge [14]. The traditional options are antihistamines, leukotriene inhibitors, and immunosuppressive agents [15]. Rapid sweat desensitization with autologous sweat has been reported in patients resistant to conventional therapy who have sweat hypersensitivity [16]. Whatever the treatment approach, [13-19] however, in some patients, cholinergic urticaria may be refractory. The aim of this study was to determine the therapeutic efficacy of different drug combinations in the treatment of cholinergic urticaria.

## Patients and Methods

### Study Population

The participants included in the study are with age range of 16 to 29 years, with cholinergic urticaria of any duration as diagnosed by physicians (allergologist). Patients were recruited from patients attending Asthma and Allergy Centers in Baghdad and Tikrit. The selected patients were divided randomly into 3 groups according to the treatment protocol. All patients completed screening before treatment. Exclusion criteria are as follows:

1. Cholinergic urticaria associated to some underlying disease (Hodgkin lymphoma/vasculitis/lupus erythematosus/hepatitis).

2. Patients under any systemic or topical medication for cholinergic urticaria and/or an inferior washout period as stated below:

• H1 antagonists, such as fexofenadine (5 days before day 0), loratadine, desloratadine, cetirizine, hydroxyzine, diphenhydramine, cyproheptadine, etc (3 days before day 0)

• H2 receptor antagonists, such as cimetidine, ranitidine, famotidine, etc (2 days before day 0).

• H1 and H2 receptor antagonists, such as doxepin (7 days before day 0).

• Leukotriene antagonists, such as zafirlukast, montelukast, etc (4 days before day 0).

• Corticosteroids, such as prednisone, methylprednisolone, etc (28 days before day 0).

• Tricyclic antidepressants, such as imipramine, amitriptiline, etc (30 days before day 0).

3. Patients with analytical values twice as high than the upper limit of normality in the following parameters: alkaline aminotransferase, aspartate aminotransferase, gama-glutamyl transferase, creatine kinase, and creatinine; and 1.5 times higher than the upper limit of normality for CK.

4. Other forms of urticaria.

5. Patients taking medications that are known to interact with CYP3A4 isozyme of cytochrome: amiodarone, carbamazepine, cyclosporine, terfenadine, glucocorticoids, phenytoin, rifampicin, macrolides (eg, erythromycin, clarythromycine) and antifungal medications (eg, ketoconazole, miconazole, fluconazole), as well as grapefruit juice.

6. Pregnant or lactating female.

Sufficient washout time was required for previous urticarial treatments (especially long-acting antihistamines and corticosteroids) before the study drugs were administered. Approval for the study was obtained from the Ethical Committee, and all patients gave their informed consent. The target population was 300 patients, 100 in each treatment group. However, 8 patients defaulted from group A, 23 from group B, and 18 from group C.

### Study Design

The recruited patients were randomized to receive one of the treatments listed below.

#### Group A

Group A includes 92 patients with age range of 16 to 29 years; they received 4 mg of chlorpheniramine maleate (histadine), half an hour before the exercise challenge, plus 5 mg of chlordiazopoxide and 2.5 mg of clindium bromide (librax), 3 times daily.

#### Group B

Group B includes 77 patients with age range of 17 to 27 years; they received 4 mg of chlorpheniramine maleate (histadine), 3 times daily, plus 25 mg of maprotiline HCl (ludiomil), once daily at night.

#### Group C

Group C includes 82 patients with age range of 19 to 29 years; they received 4 mg of chlorpheniramine maleate (histadine), 3 times daily, plus 200 mg of cimetidine (tagadine), 3 times daily.

The treatment course lasted for 6 weeks. The study comprised of 4 visits, initial and 3 follow-up visits every 2 weeks. Each patient was examined by the physician 4 times over the 6-week period. The tablets were encapsulated in a double blind fashion and sealed in envelop by a pharmacist along with the instructions sheet at the beginning of the trial. All treatments were dispensed by a third party. Medications that could interfere with the clinical evaluations and systemic or topical medication for urticaria, other than those specified in the study treatment, were not allowed during the trial. Informed patient consent was obtained before enrollment in the study.

### Patient Evaluation

Baseline assessment was performed at the beginning of the study to measure the clinical condition through complete history and physical examination. In addition, background information on personal characteristics and clinical history was also collected and family history of atopy was also recorded. Blood samples were obtained from each patient to determine white blood cell count (total and differential), packed cell volume, erythrocyte sedimentation rate, and serological test (lupus erythematosus test and Rh factor). Complete cure is defined as absence of signs and symptoms, whereas relapse is defined as recurrence of signs and symptoms.

### Safety

Safety and tolerability were assessed on the basis of the adverse events referred or changes in vital signs, physical examination findings, and electrocardiogram recorded before and at the end of treatment. Laboratory safety parameters (hematology, serum biochemistry, and urine analysis) were assessed before and after the treatment period.

### Exercise Challenge Test

It was performed for each patient and in each visit of evaluation.

### Statistical Analysis

Chi-square test and student *t *test were used for significant testing.

## Results

A total number of 251 patients with cholinergic urticaria were included in the study analysis. Of the total, 214 (85%) were male and 37 (15%) were female. All patients were adolescent or adult, with age range of 16 to 29 years.

Their characteristics at the enrollment in the study are presented in Table 1, and there are no significant differences among groups. Eosinophil count reduced after treatment in all the 3 groups. Groups B and C demonstrated a reduction in eosinophil count from the first visit, whereas in group A, the reduction was achieved in the second visit (Table 2). The reduction was more in groups B and C (P < 0.01) as compared with group A in their fourth visit (P < 0.05).

**Table 1:** Study Population Characteristics at the Time of Entry

Variable	Group A (92) Mean ± SD (Average)	Group B (77) Mean ± SD (Average)	Group C (82) Mean ± SD (Average)
Age, y	21 ± 5 (16-29)	23 ± 4 (17-27)	23 ± 3 (19-29)
WBC total count	6600 ± 120 (5100-9200)	7500 ± 800 (6000-9000)	7600 ± 960 (5900-10, 600)
Neutrophil %	61 ± 5 (52-70)	60 ± 6 (49-72)	63 ± 6 (53-74)
Lymphocyte %	29 ± 2 (21-40)	31 ± 6 (19-41)	31 ± 6 (19-42)
Eosinophil %	7 ± 1(5-9)	7 ± 1(5-8)	7 ± 1(5-9)
Monocyte %	2 ± 1(1-6)	2 ± 1(0-6)	1 ± 1(0-5)
Basophil %	0 (0-2)	0 (0-1)	0 (0-1)
ESR, mm/h	9.5 ± 3(4-20)	9 ± 2(6-12)	12 ± 5(4-36)
PCV %	42 ± 2 (38-44)	42 ± 2 (40-44)	42 ± 2 (36-42)
Platelets number	172, 000 ± 83, 900 (150, 000-180, 000)	170, 000 ± 32, 400 (148, 000-175, 000)	165, 000 ± 9750 (146, 000-177, 000)

ESR, erythrocyte sedimentation rate; PCV, packed cell volume;

**Table 2:** Eosinophil Count at Pretreatment and Posttreatment.

		Posttreatment Percent, Mean ± SD				
			
Group	Pretreatment Percent, Mean ± SD	First Visit	Second Visit	Third Visit	Fourth Visit	*P*
A	7 ± 1	7 ± 1	6 ± 1	6 ± 1	6 ± 1	< 0.05
B	7 ± 1	5 ± 1	5 ± 1	5 ± 1	4 ± 1	< 0.01
C	7 ± 1	5 ± 1	4 ± 1	4 ± 1	4 ± 1	< 0.01
*P *	> 0.05	< 0.01	< 0.05	< 0.05	< 0.01	

Itching was absent in 17.4% of patients in group A, 42.8% in group B, and 52.4% in group C (*P *= 0.0001). In addition, itching decreased in 53.3% of patients in group A, 36.4% in group B, and 30.5% in group C (*P *= 0.006) (Table 3).

**Table 3:** Clinical Response to Treatment

Variable	Group A, n (%)	Group B, n (%)	Group C, n (%)	*P*
Itching				
Increased	27 (29.3)	16 (20.8)	14 (17.1)	0.13
Decreased	49 (53.3)	28 (36.4)	25 (30.5)	0.006
Absent	16 (17.4)	33 (42.8)	43 (52.4)	0.0001
Weal				
Increased	34 (36.9)	17 (22.1)	6 (7.3)	0.0001
Decreased	28 (30.4)	12 (15.6)	10 (12.2)	0.005
Absent	31 (33.7)	48 (62.3)	66 (80.5)	0.0001
Both itching and weal absent	28 (30.4)	64 (83.1)	70 (85.4)	0.0001
Eosinophil				
Increased	0 (0)	0 (0)	8 (9.8)	0.002
Decreased	61 (66.3)	74 (96.1)	70 (85.4)	0.0001
Same	31 (33.7)	3 (3.9)	4 (4.8)	0.0001

Weal was absent in 33.7% of patients in group A, 62.3% in group B, and 80.5% in group C (*P *= 0.000). However, weal reduced in 12.2% of patients in group C, 15.6% in group B, and 30.4 in group A (*P *= 0.005). Both weal and itching were absent in 30.4% of group A, in 83.1% of group B, and in 85.4% of group C (*P *= 0.0001).

Eosinophil count reduction was higher in group B (96.1%) as compared with group C (85.4%) and group A (66.3%), with highly significant (*P *= 0.0001) differences. Table 4 shows the comparison between the 3 groups with regard to the presence of signs and symptoms and eosinophil count. The absence of signs and symptoms was demonstrated in 55 patients (67.1%) in group C, 36 patients (44.2%) in group B, and 3 patients (3.3%) in group A in their first visit (*P *= 0.0001). In addition, eosinophil count was lower in groups B and C than that in group A (*P *= 0.0001), whether the patients were with signs and symptoms or without (Table 4). However, in groups B and C, eosinophil count was higher (6%) in group of patients with signs and symptoms than that without (5%). In visits 2, 3, and 4 the same pattern was demonstrated for eosinophil count.

**Table 4:** Comparison of Eosinophil Count in Patients With Signs and Symptoms of Cholinergic Urticaria and Those Without

Variable		Group A	Group B	Group C	*P*
First	No signs and symptoms				0.0001
visit	n (%)	3 (3.3)	34 (44.2)	55 (67.1)	
	Eosinophil %	7	5	5	
	Signs and symptoms				0.01
	n (%)	89 (96.7)	43 (55.8)	27 (32.9)	
	Eosinophil %	7	6	6	
Second	No signs and symptoms				0.01
visit	n (%)	20 (21.7)	53 (68.8)	64 (78)	
	Eosinophil %	5	4	4	
	Signs and symptoms				0.01
	n (%)	72 (78.3)	24 (31.2)	18 (22)	
	Eosinophil %	7	6	6	
Third	No signs and symptoms				> 0.05
visit	n (%)	25 (27.2)	60 (77.9)	65 (79.3)	
	Eosinophil %	4	4	4	
	Signs and symptoms				0.01
	n (%)	67 (72.8)	17 (22.1)	17 (20.7)	
	Eosinophil %	6	6	5	
Fourth	No signs and symptoms				0.01
visit	n (%)	28 (30.4)	64 (83.1)	70 (85.4)	
	Eosinophil %	4	4	3	
	Signs and symptoms				0.01
	n (%)	64 (69.6)	13 (16.9)	12 (14.6)	
	Eosinophil %	6	6	5	

The complete control in the first visit was higher in group C (67.1%) than in group B (44.2%) and group A (3.3%) (*P *= 0.0001). The same pattern of response to treatment was demonstrated in other follow-up visits (Table 5). In addition, the complete control was with high significant differences between first, second, third, and fourth visits for groups A and B (*P *< 0.0001) and borderline significant for group C (*P *= 0.04). This is because that response to treatment was initiated earlier in group C as compared with groups A and B.

**Table 5:** Complete Control in Different Groups

Visit	Group A, n (%)	Group B, n (%)	Group C, n (%)	*P*
First	3 (3.3)	34 (44.2)	55 (67.1)	0.0001
Second	20 (21.7)	53 (68.8)	64 (78)	0.0001
Third	25 (27.2)	60 (77.9)	65 (79.3)	0.0001
Fourth	28 (30.4)	64 (83.1)	70 (85.4)	0.0001
*P*	0.0001	0.0001	0.04	

The frequency of relapse was higher in group A (89%) as compared with group B (68.4%) and group C (23.5%) (*P *< 0.0001) (Table 6). Adverse effects varied between the groups (Table 7), and the variation was statistically significant (*P *< 0.0001). For group A, the most common side effect was dry mouth (82.6%), followed by irritability (59.8%), drowsiness (37%), and blurred vision (27.2%). In group B, the most common side effect was weight gain (81.8%), followed by drowsiness (74%), night mares (64.9%), dry mouth (61%), muscle weakness (48.1%), and gastrointestinal tract disturbance (42.8%). In group C, the most common side effect was gynecomastia (62.2%) and weight gain (59.7%), followed by myalgia (47.6%), diarrhea (46.3%), and muscle weakness (44.2%) (Table 7).

**Table 6:** Frequency of Relapse After Cessation of Treatment

Group	Relapse Number	Relapse Percent
A	32/36	89
B	39/57	68.4
C	16/68	23.6

*P *= 0.0001.

**Table 7:** Frequency of Side Effects

	No. Side Effects (%)		
	
Side Effect	Group A	Group B	Group C
Dry mouth	76 (82.6)	47 (61.0)	0 (00.0)
Irritability	55 (59.8)	17 (22.1)	0 (00.0)
Drowsiness	34 (37.0)	57 (74.0)	0 (00.0)
Blurred vision	25 (27.2)	26 (33.8)	0 (00.0)
GIT disturbance	15 (16.3)	33 (42.8)	0 (00.0)
Headache	15 (16.3)	10 (13.0)	0 (00.0)
Difficulty in micturition	12 (13.0)	0 (00.0)	0 (00.0)
Hypotension	9 (9.80)	7 (9.10)	16 (19.5)
Weight gain	6 (6.50)	63 (81.8)	49 (59.7)
Diarrhea	3 (3.30)	0 (00.0)	38 (46.3)
Night mares	0 (00.0)	50 (64.9)	25 (30.5)
Muscle weakness	0 (00.0)	37 (48.1)	34 (44.2)
Myalgia	0 (00.0)	7 (9.10)	39 (47.6)
Arthralgia	0 (00.0)	4 (5.20)	10 (12.2)
Gynecomastia	0 (00.0)	0 (00.0)	51 (62.2)
Tingling of extremities	0 (00.0)	0 (00.0)	4 (4.90)

## Discussion

Effective therapeutic approaches for the treatment of cholinergic urticaria are not well established. Thus, this study was conducted as double blind but not placebo controlled to evaluate therapeutic efficacy of different treatment combination in cholinergic urticaria. The study indicated that combination used in group C was the most effective treatment combination. However, the present study shows that complete control was demonstrated only in 30.4% of patients (group A) receiving 4 mg of chlorpheniramine maleate, half hour before the exercise, plus chlordiazopoxide (5 mg) and clindium bromide (2.5 mg) tablets, 3 times daily. However, complete control was achieved in higher rate (83.1%) in patients (group B) receiving 4 mg of chlorpheniramine maleate (histadine), 3 times daily, plus 25 mg of maprotiline HCl (ludiomil), once daily at night. Furthermore, the complete control was 85.4% in patients (group C) receiving 4 mg of chlorpheniramine maleate (histadine) plus 200 mg of cimetidine (tagadine), 3 times daily. The differences in response rate were statistically highly significant between group A in one hand and groups B and C in another hand. In addition, the difference in complete control between groups B and C was statistically significant. Thus, a better therapeutic response is induced by drug combinations used in group C [4 mg of chlorpheniramine maleate (histadine), 3 times daily, plus 200 mg of cimetidine (tagadine), 3 times daily].

The relapse rate was higher (89%) in group of patients receiving chlorpheniramine maleate plus chlordiazopoxide and clindium bromide as compared with group of patients receiving chlorpheniramine maleate plus maprotiline HCl (68.4%) and group of patients receiving chlorpheniramine maleate plus cimetidine (23.5%). The above differences in relapsing rate were statistically highly significant.

From the above findings we conclude that combination of chlorpheniramine maleate with cimetidine was the effective treatment combination for cholinergic urticaria. This effect may be due to combination of both H1 and H2 antagonists.

The complete control was significantly (*P *< 0.0001) different for group C (67.1%) as compared with group B (44.2%) and group A (3.3%) in their first visit. These findings indicate that treatment of cholinergic urticaria with combination of H1 and H2 antagonists exerted its therapeutic effect earlier than other combinations.

In the literature, there is only one clinical trial published that evaluated cetirizine as treatment for cholinergic urticaria in 24 patients [20]. Their study reported that cetirizine (10 or 20 mg), for 3 weeks' period, was satisfactory for the treatment of cholinergic urticaria. However, there are many successful treatments of selected cases of cholinergic urticaria in literature with different treatments. Volcheck and Li[7] reported cases of exercise-induced urticaria treated with antihistamines (H1 antagonist) only and for open period. In addition, Alexander[8] used a long acting non sedating antihistamines taken 1 hour before the exercise help in preventing exercise -induced urticaria and they used prednisolone as an alternative for antihistamine if failed to give response.

Kaplan et al[6] recommended H1 antagonist for prophylactic and acute therapy for cholinergic urticaria. They used different classes of H1 antagonist and found that hydroxyzine is preferred for cholinergic urticaria. Lewis and Erffmeger[21] reported 3 cases of exercise-induced urticaria treated with hydroxyzine with good response. Feinberg and Toner17 reported a successful treatment of disabling cholinergic urticaria with combination of cetirizine, montelukast, and propranolol.

Beta-blockers, such as propranolol, have been reported to be useful in cholinergic urticaria [13]. Both topically applied benzoyl scopolamine and oral scopolamine butylbromide may be helpful in blocking the appearance of cholinergic urticarial lesions after challenge[14]. Traditional options are antihistamines, leukotriene inhibitors, and immunosuppressive agents [15]. However, in some patients cholinergic urticaria may be refractory. Rapid desensitization with autologous sweat has been reported in patients resistant to conventional therapy who have sweat hypersensitivity [16]. La Shell and England[11] treated a case of severe, refractory cholinergic urticaria with danazol, with significant improvement in the control of the urticaria.

Furthermore, Metz et al[18] reported successful treatment of cholinergic urticaria with anti-IgE therapy. However, Sabroe[22] reported that omalizumab, a monoclonal IgG anti-IgE antibody, which is successfully used in one case of cholinergic urticaria, was not effective in control of severe cholinergic urticaria. Although, the effectiveness of each therapy mentioned above varies and does not reach the standard agreement [23].

Cholinergic urticaria has well-described, characteristic clinical presentations, yet the precise pathological mechanism remains incompletely understood [23]. Recent reports have demonstrated that subcutaneous injection of cholinergic agents induce sweating and hives development in patients with cholinergic urticaria and that the symptoms of cholinergic urticaria are inhibited by previous atropinization of the skin[24]; recent studies indicated that mast cells express muscarinic cholinergic receptor, which is a responsible cholinergic receptor for sweating [25]. In addition, acetylcholine triggers rat mast cell degranulation [26]. Thus, the above findings collected together may suggest a role for acetylcholine in pathogenesis of cholinergic urticaria.

Serum histamine levels are elevated in some patients with cholinergic urticaria, [3] suggesting a role for histamine in cholinergic urticaria pathogenesis. Furthermore, treatment of cholinergic urticaria with antihistamines is of limited successful response in most cases of cholinergic urticaria. This may suggest that histamine plays a minor role in cholinergic urticaria pathogenesis and additional mediators may be involved [3,7]. Nakamizo et al[23] proposed a variety of cholinergic urticaria pathogenesis, which in turn lead to suggestion of existence of several clinical subtypes. Thus, the presence of these clinical subtypes may influence the response to different types of treatment.

The limitation of this study is that the upper limit efficacy for H1 antagonists is not determined and may be greater than 3 days. Thus, adding a fourth tablet or more may be just as good as adding cimetidine. For example, in cold urticaria, 4 levocetirizine tablets were better than 3, and for cold urticaria and cholinergic urticaria, the dose of hydroxyzine used for severe cases equaled 6 cetirizine tablets.

In conclusion, this study indicated that combination of H1 and H2 antagonists was more effective based on complete control of cholinergic urticaria with lower relapsing rate. However, a future placebo-controlled clinical trial taking in consideration higher H1 antagonists than we used is warranted.

## Competing interests

The authors declare that they have no competing interests.
